# Orphan drugs expenditure in the Netherlands in the period 2006–2012

**DOI:** 10.1186/s13023-014-0154-0

**Published:** 2014-10-11

**Authors:** Tim A Kanters, Adri Steenhoek, Leona Hakkaart

**Affiliations:** Institute for Medical Technology Assessment, Department of Health Policy & Management, Erasmus University Rotterdam, Burgemeester Oudlaan 50, P.O. Box 1738, 3000DR Rotterdam, The Netherlands

**Keywords:** Orphan drugs, Uptake, Budget impact, Reimbursement

## Abstract

**Background:**

The relatively low budget impact of orphan drugs is often used as an argument in reimbursement decisions. However, overall, the budget impact of orphan drugs can still be substantial. In this study, we assess the uptake and budget impact of orphan drugs in the Netherlands.

**Methods:**

We examined the number of orphan drugs, the number of patients and budget impact of orphan drugs in the Netherlands in the period 2006 to 2012, both for inpatient and outpatient orphan drugs. Budget impact was provided in absolute numbers and relative to total pharmaceutical spending.

**Results:**

The number of orphan drugs and patients treated increased substantially over the period studied. Overall, budget impact increased substantially over a period of six years, both in absolute terms (326% increase) as well as relative to total pharmaceutical spending (278% increase). Growth rates decreased over time. In 2012, 17% of available drugs had an individual budget impact of more than €10 million per year.

**Conclusions:**

Individual budget impact of orphan drugs is often limited, although exceptions exist. However, in total, the budget impact of orphan drugs is considerable and has grown substantially over the years. This could potentially influence reimbursement decisions for orphan drugs in the future.

## Background

The introduction of orphan drug legislation in various jurisdictions has played an important role for the development of orphan drugs; since European legislation was passed in 2000, 73 drugs for orphan indications were licensed [1]. However, on a national level this also poses policy makers for difficult decisions concerning reimbursement, as illustrated by examples from the United Kingdom and the Netherlands [2,3]. On the one hand, therapies are developed for diseases that, next to being rare, are life-threatening and chronically debilitating by definition [4]. On the other hand, orphan drug prices can be enormous, as pharmaceutical companies recoup their investments in research and development on the small patient population. The high prices of orphan drugs place decision makers for a difficult task; cost-effectiveness ratios for these drugs are often very high, implying that the money spent on orphan drugs might be spend more efficiently in other disease areas. However, there are also arguments in favor of granting reimbursement to orphan drugs. Ethical considerations, the lack of alternative treatments and severity of the disease all apply to the case of orphan drugs [5].

Budget impact is yet another criterion used in reimbursement decisions [6,7]. For orphan drugs, the impact on the pharmaceutical budget is limited due to the small number of patients, let alone the impact on the entire healthcare budget. Although this proposition holds for individual orphan drugs, the combined budget impact of all available orphan drugs might be considerable, especially as the number of orphan drugs on the market is still increasing. Various studies in several European countries have shown the increasing share of pharmaceutical spending that is spent on orphan drugs [8-11]. Moreover, these studies have shown that differences between countries exist. In this study we assess the uptake and overall budget impact of orphan drugs in the Netherlands. As such, the results of this study can be useful for Dutch policymakers, as well as for validating results from earlier studies in other countries and providing a benchmark for countries were these studies have not been performed yet.

## Methods

In the Netherlands, outpatient and inpatients orphan drugs are financed differently. Firstly, outpatient drugs are financed through the common Drug Reimbursement System (GVS). Outpatient cancer orphan drugs were transferred to the specialist drugs list in 2013. Secondly, inpatient drugs were financed through a specific policy rule on orphan drugs (until 2012) and since 2012 through an “add-on” diagnosis treatment combination (DBC). We examined the number of orphan drugs in the Netherlands, the number of patients using them and the orphan drugs’ budget impact.

### Outpatient drugs

For outpatient drugs we examined the Drug Information Project (GIP) database hosted by the Health Care Insurance Board (CVZ) [12]. The GIP database is publicly accessible and contains information on the use and costs of medicines and medical devices. With respect to medicines, the database contains detailed information on number of patients, usage and expenditures. Some orphan drugs were also prescribed for non-orphan indications. For these drugs, only data related to orphan indications was taken into account. Prices were calculated by dividing drug’s budget impact by the number of defined daily doses multiplied by 365.25 to get price of treatment per year.

### Inpatient drugs

From 2006 to 2012, inpatient drugs were financed through a specific policy rule on orphan drugs. When applying for inclusion on the policy rule, pharmaceutical companies had to make predictions on the number of users, prices and budget impact of the drug. Furthermore, after a reimbursement period of four years, pharmaceutical companies had to submit an outcomes research report with, among other aspects, clinical outcomes, observed number of users and budget impact. The primary sources of information for the current study were the outcomes research reports and the policy rule applications. When outcomes research reports and policy rule applications were not available, data on an aggregated level from the Monitor Expensive Drugs were used [13].

### Analyses

Budget impact was expressed in euros and as a percentage of total pharmaceutical spending. Total pharmaceutical spending in the Netherlands (orphan drugs and non-orphan drugs) was derived from the GIP database for outpatient drugs. Total pharmaceutical spending on inpatient drugs were derived from FarmInform [14]. Total pharmaceutical spending resulted from the summation of inpatient and outpatient drugs spending.

In addition to the analyses with regard to all orphan drugs combined, characteristics are provided for the five orphan drugs ranked highest with regard to the number of patients treated, the price of the orphan drug and the individual drug’s budget impact.

For some inpatient drugs, data was not available for all years. In these cases, the last observation was used for later years, implying a conservative scenario. As a sensitivity analysis, the individual drugs’ growth rate for the last known year was used for extrapolation instead.

## Results

Table 1 shows the uptake of orphan drugs and evolution of orphan drug spending over time in the Netherlands. Considering uptake of orphan drugs, both the number of drugs and the number of patients almost quadrupled over the studied period. Both factors contributed to the increase in budget impact of orphan drugs over time, both for inpatient and outpatient drugs. For inpatient drugs, the number of patients was relatively stable for each drug. For outpatient drugs, the number of users increased substantially; with an average growth of 168 patients over the study period.

**Table 1:** **Uptake and budget impact of orphan drugs in the Netherlands**

	**2006**	**2007**	**2008**	**2009**	**2010**	**2011**	**2012**
*Number of orphan drugs*							
Outpatient	9	15	18	22	25	30	32
Inpatient	2	7	8	9	11	11	11*
Total	11	22	26	31	36	41	43
*Number of patients treated with orphan drugs*							
Outpatient	2,149	3,457	4,410	6,024	7,621	8,250	9,226
Inpatient	40	146	215	469	531	536	536*
Total	2,189	3,603	4,625	6,493	8,152	8,786	9,762
*Budget impact of orphan drugs (millions)*							
Outpatient	€52.7	€68.7	€97.8	€118.1	€141.6	€156.2	€175.2
Inpatient	€8.5	€29.2	€60.7	€74.6	€84.3	€85.1	€85.1*
Total	€61.2	€97.9	€158.6	€192.7	€225.9	€241.4	€260.4

*For inpatient drugs, figures for 2012 were assumed equal to 2011.

For inpatient drugs, prices were constant over the time period studied. The average annual treatment costs for inpatient drugs was €255,615 (SD = 223,306). The average annual treatment costs for outpatient drugs was €40,679 in 2012 (SD = 45,283). For outpatient drugs, some variation was observed over the study period, but for most drugs price changes were modest. For 39.3% of the inpatient drugs, prices decreased with more than 2%. In contrast, prices increased with more than 2% for 17.9% of drugs. On average, drug prices slightly decreased over time (−1.2%).

Table 1 further shows that over a period of seven years, the total expenditure on orphan drugs quadrupled. The growth rate was decreasing over time; from 60.1% in the period 2006 to 2007 to 7.9% in 2011–2012. The absolute growth in budget impact was largest for outpatient drugs. The relative growth in budget impact over seven years was largest for inpatient drugs.

**Figure 1:**
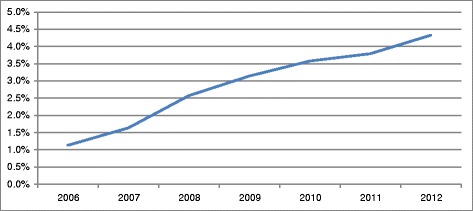
**Budget impact of orphan drugs as a proportion of total drug spending.**

Figure 1 provides the development of budget impact of orphan drugs over time as a percentage of total pharmaceutical spending. The proportion of total pharmaceutical spending spent on orphan drugs almost quadrupled from 1.1% in 2006 to 4.2% 2012. The relative growth rate decreased over time. Total pharmaceutical spending increased with 12.6% in the period 2006–2012.

Table 2 shows the orphan drugs that were most often used in 2012. In 2012, a total of 1,485 patients received imatinib. The most-often used inpatient drug was trabectedin (Yondelis®, 240 patients). More than 60% of all orphan drug receiving patients in the Netherlands received one of the five most-used drugs, which are listed in Table 2. Lenalidomide (Revlimid®) was the drug with the biggest increase in the number of users over time; from 79 users in 2007 to 1,089 in 2012.

**Table 2:** **Orphan drugs with highest number of users, highest prices and highest budget impact (2012)**

**Rank**	**INN**	**Trade name**	**Setting**	**Patients**	
1	Imatinib	Glivec®	Outpatient	1,485	
2	Lenalidomide	Revlimid®	Outpatient	1,089	
3	Sildenafil	Revatio®	Outpatient	1,052	
4	Thalidomide	Thalidomide Cellgene®	Outpatient	1,030	
5	Bosentan	Tracleer®	Outpatient	901	
Rank	INN	Trade name	Setting	Cost/Patient	
1	Galsulfase	Naglazyme®	Inpatient	€600,000	
1	Idursulfase	Elaprase®	Inpatient	€600,000	
3	Alglucosidase alfa	Myzoyme®	Inpatient	€474,857*	
4	Eculizumab	Soliris®	Inpatient	€358,000	
5	Aldurazyme	Laronidase®	Inpatient	€300,000	
Rank	INN	Trade name	Setting	Budget impact (millions)	Cumulative budget impact 2006–2012 (millions)
1	Alglucosidase alfa	Myozyme®	Inpatient	€40.3	€203.4
2	Imatinib	Glivec®	Outpatient	€36.4	€251.2
3	Lenalidomide	Revlimid®	Outpatient	€36.2	€127.4
4	Bosentan	Tracleer®	Outpatient	€23.0	€132.4
5	Pegvisomant	Somavert®	Outpatient	€14.3	€72.5

INN = International Nonproprietary Names; *weighted average of infantile patients (€706,666/patient) and adult patients (€422,314/patient).

Yearly treatment costs exceeded €100,000 per patient for seven inpatient drugs (63.6%) and for two outpatient drugs (6.3%). The orphan drugs with the highest per patient prices are provided in Table 2. Velaglucerase alfa (VPRIV®) was the highest priced inpatient drug, with annual per patient costs of approximately €200,000 in 2012. Tafamidis (Vyndaqel®) was the only other inpatient drug with annual costs over €100,000. Yearly costs for three (five) drugs were lower than €2,000 (€3,000).

Table 2 also shows the orphan drugs with the highest budget impact in 2012. Seven out of 41 orphan drugs (17%) had an individual budget impact exceeding €10 million; the budget impact of 18 outpatient and nine inpatient drugs was more than €1 million in 2012. The drug with the largest budget impact in 2012 was alglucosidase alfa. Together, the five drugs with the largest budget impact accounted for 57.7% of the total budget impact of orphan drugs. Four drugs (one inpatient) had a cumulative budget impact of more than €100 million over the study period. Imanitib’s cumulative budget impact exceeded €250 million.

### Sensitivity analysis

For missing data for inpatient drugs, we used the last observed budget impact. For drugs with a growing budget impact over time (majority of the 11 inpatient drugs) this might have led to an underestimation of the budget impact. Extrapolation of the last observed growth rates for individual drugs’ resulted in an additional budget impact of €14 million in 2012. In this analysis, 4.6% of total pharmaceutical spending would be spent on orphan drugs in 2012. In contrast to the base case analysis, the growth rate would be constant over time.

## Discussion

In this study, we showed that the budget impact of orphan drugs in the Netherlands increased substantially over time, both as a proportion of total drug spending as well as in absolute terms. The growth in budget impact was explained by the increasing number of orphan drugs available and the increasing number of patients receiving the drugs.

The proportion of total pharmaceutical spending on orphan drugs in the Netherlands for 2007 was 1.6%; similar to proportional spending in Spain (2.0%), Germany (2.1%) Italy (1.5%) and France (1.7%); and higher than in the UK (1.0%) [9]. For later years, budget impact in the Netherlands was higher than in Belgium (1.9% in 2008, compared to 2.6% in the Netherlands), Sweden and France (respectively 2.5% and 3.1% in 2012, compared to 4.2% in the Netherlands) [8,11]. It should be noted that comparing our results to studies from other countries is difficult due to transferability issues: inter-country differences with respect to reimbursement decisions of (individual) orphan drugs and prices of orphan drugs exist [Kanters et al: Factors affecting reimbursement decisions on 11 high-priced inpatient orphan drugs, submitted]. Furthermore, total pharmaceutical spending differs between countries, which could also affect the proportion spent on orphan drugs.

Two earlier studies have used a model to predict future proportion of pharmaceutical spending spent on orphan drugs [10,11]. Schey et al. [10] predicted the proportion of total pharmaceutical spending spent on orphan drugs to decrease from 2016 onwards [10]. An important element in their model was the assumption that orphan drug prices would decrease as a consequence of competition. Until now, the generic orphan drugs market did not expand, and it can be questioned whether the orphan drug market is attractive enough for generic companies to enter the field of rare diseases. More recently, Hutchings et al. [11] forecasted that the proportion of total drug spending on orphan drugs in Sweden and France would increase until 2018, after which a steady state would be reached [11]. More research is needed to establish whether the steady state would actually be achieved and how these figures apply to other countries.

Our results show similarities with other studies over time increasing budget impact relative to total pharmaceutical spending but with decreasing growth rates [10,11]. Over time, growth rates were decreasing. This might be explained by saturation of the target population; most eligible patients receive the available drug. Saturation might be especially high for these orphan drugs as alternative treatments are often non-existent.

### Limitations

The time period in this study was limited to a period of seven years. Even in this relatively short time period, we observed a substantial growth in orphan drugs spending. A longer study period would be needed to investigate whether the growth rates continue to decrease, and if so whether a negative growth rate, i.e. a decreasing budget impact, will be observed in the future. Due to a change in financing outpatient cancer drugs since 2013, the analyses could not be extended to 2013. Remarkably, when a subsample of outpatient drugs that were available for 2013 (n = 26) was analyzed, a decrease (−6.0%) of the number of patients treated was found. Despite the decrease in the number of patients treated, an increase in the total budget impact is observed. The growth rate of the budget impact relative to the previous year was modest (4.8%). Two new outpatient orphan drugs entered the Dutch market in 2013.

To predict the future budget impact of orphan drugs in the Netherlands, detailed information is needed on availability of individual orphan drugs in the Netherlands, number of patients using these drugs, and prices of orphan drugs in the Netherlands, also in relation to generic competition for orphan drugs. This information is yet unavailable for the Netherlands. Further research on these aspects is needed before a prediction of the future budget impact of orphan drugs in the Netherlands can be made.

### Implications

The combined budget impact of orphan drugs is substantial and increasing. Budget impact for individual orphan drugs might be limited (although 17% of orphan drugs had an individual budget impact exceeding €10 million). However, policy makers should acknowledge the increasing budget impact of all orphan drugs. The small budget impact might therefore not be a valid argument in discussions on reimbursement of orphan drugs, especially as the number of orphan drugs continues to grow and hence so will their budget impact.

## Conclusions

The number of available orphan drugs and the number of patients receiving these orphan drugs have substantially increased over the last years in the Netherlands. Accordingly, the budget impact associated with these orphan drugs have increased, both in absolute terms as well as compared to the total pharmaceutical spending.
